# ‘A lifebuoy’ and ‘a waste of time’: patients’ varying experiences of multidisciplinary pain centre treatment- a qualitative study

**DOI:** 10.1186/s12913-019-4876-5

**Published:** 2019-12-30

**Authors:** Torunn Hatlen Nøst, Aslak Steinsbekk

**Affiliations:** Department of Public Health and Nursing, Norwegian University of Science and Technology, 7491 Trondheim, Norway

**Keywords:** Chronic pain, Pain Centre, Multidisciplinary, Qualitative

## Abstract

**Background:**

The recognition of chronic pain as a biopsychosocial phenomenon has led to the establishment of multidisciplinary pain treatment facilities, such as pain centres. Previous studies have focussed on inpatient, group-based or time-limited multidisciplinary pain programmes. The aim was to investigate variation in patients’ experiences of attending individual outpatient multidisciplinary treatment at pain centres in Norway.

**Methods:**

This was a qualitative study using semi-structured individual interviews with 19 informants. The informants were recruited among persons who after referral by their general practitioners 12 months prior had attended multidisciplinary pain treatment at a pain centre. The data were analysed thematically using systematic text condensation.

**Results:**

The informants had received different treatments at the pain centres. Some had undergone only one multidisciplinary assessment in which a physician, a psychologist and a physiotherapist had been present, whereas others had initially been to a multidisciplinary assessment and then continued treatment by one or more of the professionals at the centre. Their experiences ranged from the pain centre as being described as a lifebuoy by some informants who had attended treatment over time, to being described as a waste of time by others who had only attended one or two multidisciplinary sessions. Prominent experiences included being met with understanding and a perception of receiving the best possible treatment, but also included disappointment over not being offered any treatment and perceiving the multidisciplinary approach as unnecessary.

**Conclusions:**

There were large variations in the informants’ experiences in the pain centres. The findings indicate that the pain centres’ multidisciplinary approach can represent a new approach to living with chronic pain but may also not provide anything new. Efforts should be devoted to ensuring that the pain centres’ multidisciplinary treatment approach is aligned with their patients’ actual needs.

## Background

Chronic pain is a major health problem for a substantial proportion of the population [1] and is thus a significant public health issue [2, 3]. Studies performed in Europe have shown that chronic non-cancer pain affects between 10 and 30% of the adult population [4, 5], and the estimation for Norway is that about one-third of the population suffers from chronic pain [6]. The consequences following the condition are extensive, including high healthcare utilisation, reduced work participation, increased disability payments and lost tax revenue [2, 7]. In addition, the individuals carry a significant burden related to physical, psychological and social consequences [7, 8].

Despite many available pain treatments, the complexity of pain makes it difficult to treat, implying it may be challenging to help people with persistent pain to achieve an improved quality of life [9]. For instance, it has been stated that only a 30% reduction of pain is achieved in about half of treated patients [10] and that participation in treatments may have more subjective impact than objective outcomes [11].

The impact and the complexity of chronic pain have given way to treating chronic pain as a biopsychosocial phenomenon [12, 13]. This has led to a focus on multidisciplinary pain treatment approaches as well as to the establishment of multidisciplinary pain treatment facilities where interventions target a variety of factors simultaneously [10, 14]. According to The International Association for the Study of Pain (IASP), multidisciplinary pain centres are defined as being staffed by a variety of health care professions with expertise in pain management, including physicians, nurses, mental health professionals and physical therapists [15]. By working together, the professionals are expected to effectively assess and treat any pain problem [14].

Most multidisciplinary pain centre facilities, including the four in Norway, have been established as tertiary care facilities [16, 17]. They mainly provide services for those not receiving adequate pain alleviation in primary care or by organ-specific specialists in secondary care. This means that the individuals attending pain centres are mostly those not helped by other pain treatment services provided by primary or secondary care [16].

Several studies have investigated the effectiveness of multidisciplinary pain treatments. Some have found them to be effective, although questions remain regarding which treatment components are of importance and for whom [18, 19]. Few studies have explored patients’ experiences of attending multidisciplinary pain facilities. Those that have, showed that the learned techniques were useful even years after participation [20] and that patients perceived that the professionals empowered them to take responsibility for their daily lives, including their health [21]. Moreover, a recent qualitative evaluation of an interdisciplinary 10-week chronic pain programme showed that the spectrum of impact of the intervention ranged from whole life change to no change at all [22]. Notably, most studies have focussed on inpatient, group-based or time-limited multidisciplinary pain programmes. Thus, there is little knowledge related to how it is experienced to attend individual, outpatient multidisciplinary treatment at tertiary care pain centres.

The aim of the current study was therefore to investigate variation in patients’ experiences of attending individual outpatient multidisciplinary treatment at one of the four tertiary care pain centres in Norway.

## Methods

A qualitative study with semi-structured individual interviews was conducted. The study was done in conjunction with a larger evaluation of the pain centres in Norway financed by the Ministry of Health and Care services. All the study interviews were conducted in November 2018.

### Setting

The four Norwegian pain centres, one at each of the regional university hospitals, offer outpatient services to people suffering from pain, regardless of diagnosis. Potential patients are referred by general practitioners (GPs) or medical specialists. The national guidelines for the pain centres, including criteria for whom to grant treatment, are given by the government to ensure services of equal quality regardless of which pain centre individuals attend [23].

All referrals to the pain centres are evaluated according to the national guidelines by an admission team comprising pain physicians, clinical psychologists and physiotherapists [23, 24]. The pain centres care for a heterogenous group of patients, which means that they have to respond to different treatment needs. Common for the pain centres’ treatment is thus the multidisciplinary approach, which typically comprise a multidisciplinary assessment prior to outpatient treatment by one or more of the professionals at the pain centre followed by a final session in which all involved staff are present [24]. The patients are requested to complete a comprehensive pain questionnaire before coming to the pain centre.

### Informants and recruitment

Eligible informants were persons referred by their GPs to one of the Norwegian pain centres who were registered in the patient administration system as having begun multidisciplinary pain treatment approximately 12 months prior. To obtain data that represented the variations in patient experiences from attending Norwegian pain centres, the aim was to have variation in pain centres (four to five informants from each), as well as in age, gender and pain experience.

The first 10–20 patients who the staff identified to meet the inclusion criteria were sent an invitation that included information on the intentions of the study, a consent form and a prepaid envelop. When signed consent was returned, the pain centre provided the name and phone number to the first author who contacted them to make an appointment. A reminder text message was sent by the pain centre staff if a response was not received after 2–3 weeks. Recruitment continued until 19 informants were interviewed because we then considered to have sufficient data to answer the research question.

### Data collection and interview guide

The first author conducted all interviews, either at the pain centre or by telephone, based on the choice of the informants. The interviews lasted between 25 and 50 min (mean duration 34 min). Notes and reflections were written down immediately after each interview. The interview guide was semi-structured with open-ended questions to allow the informants to speak freely (see Additional file 1). The guide was developed for this study, based on the research question, previous studies and discussions among the authors who have experiences from similar studies. To determine whether the interview guide required alterations, the authors evaluated the recordings of the first two interviews, but no changes were made.

The main question in the interview guide was ‘Can you talk about your experiences regarding the treatment you received at the pain centre?’. This was followed by an introduction of topics concerning expectations of the pain centre’s treatment, whether they found the pain centre different from other treatments they had attended, whether there were any changes in how they managed their pain and how they viewed their futures.

### Data analysis

All interviews were audio recorded. They were repeatedly listened to by the first author who took notes and transcribed the most important parts that were used during the analysis process.

The data were analysed using systematic text condensation, which is a descriptive thematic cross-case analysis strategy involving an iterative four-step analysis procedure [25]. In the first step, the authors worked to gain an overall impression of the data and identified six preliminary themes. In the second step, the first author systematically reviewed all the interviews to identify meaning units relevant for the research question. The meaning units were coded, classified and sorted into code groups related to the preliminary themes which were discussed among the authors. It was found that classifying the results alongside a timeline from time of the referral, via the pain centre treatment and to the time of the interview, provided a coherent presentation of the data. The preliminary themes were adjusted to fit with this time- and trajectory-oriented approach.

In the third step, the first author performed a systematic abstraction of meaning units within each of the themes, reducing the content into a condensate that maintained the informants’ responses. The authors had discussions on the condensates resulting in adjustments and renaming of the themes. In the final step, the content of the condensates was synthesised into generalised descriptions and concepts, while ensuring that the result still reflected the original context.

The first author identified illustrative citations, which were translated by the first author and validated by the co-author. MindManager [26] was used as the systematization tool during the analyses. To expose the data for different views and perspectives, preliminary results were discussed several times with an extended research group on patient education and participation at the university.

## Results

Of the 19 interviews, 15 were performed by telephone. The informants were 11 women and eight men, with a mean age of 47 years (range 24–65 years) (Table 1). On average, they had experienced pain for 10 years (range 2–37 years). For most informants, pain was related to musculoskeletal and joint diseases (53%).

**Table 1:** Characteristics of the informants (*n* = 19)

Characteristic	Number
Gender	
Female	11
Male	8
Age	
< 35 years	5
35–50 years	5
51–60 years	6
61 years or more	3
Living status	
Living with family members	14
Living alone	5
Working status	
Working part or full time	4
Not working	15
Pain duration	
1–5 years	8
6–9 years	4
10 years or more	7
Main reason for pain	
Musculoskeletal and joint pain	10
Neuropathy and nerve damages	5
Abdominal pain	1
Other diseases or injuries	3

The informants had received different types and lengths of treatments at the pain centres (Table 2). Most informants (14 of 19) had been to at least one multidisciplinary assessment in which a physician, a psychologist and a physiotherapist had been present. The treatment period varied from one day to over one year, and time since the last contact with the pain centre ranged from two weeks to 1 ½ years. Two informants were still receiving treatment.

**Table 2:** Overview over the informants’ treatment at the pain centre (n = 19)

	Initially and/ or closing session			Follow-up session		
Treatment/ activity	None	Once	Twice	1–6 months	7–12 months	> 12 months
Multidisciplinary sessions ^*^	5	11	3	0	0	0
Separate sessions psychologist	5	10	1	2	0	1
Separate sessions physiotherapist	6	10	1	1	0	1
Separate sessions physician	7	8	0	2	2	0
Separate sessions nurse	18	0	0	0	1	0
Procedures e.g., nerve block, spinal stimulator	17	2	0	0	0	0

^*^ = sessions where multidisciplinary professions were concurrently present

Prior to the pain centre referral, most informants had used multiple healthcare services due to pain, including regular contact with their GPs. Examples of services used were physiotherapy, physical activity support and rehabilitation centres in addition to medical specialists such as rheumatologists, neurosurgeons, neurologists and specialists in internal medicine.

The findings were categorised into the following themes: expectations of, experiences from and changes after attending the pain centre.

### Expectations of attending the pain Centre

Some of the informants spontaneously said they did not have any expectations of attending the pain centre. Some had only been a patient there because their GPs did not know what else to do for them. Others said they had vague expectations due to a lack of knowledge about the service. Other informants had received information regarding what to expect of the pain centre from their GPs, and yet others had searched the Internet and social media for information and consequently had some knowledge of the pain centre treatment procedures. Still, they said they had few expectations of attending the pain centre.

When asked to elaborate, informants discussed areas in which they hoped to receive help. Some said that they had no expectations that the pain centre treatment would alleviate their pain, as no other pain treatment previously had been efficient; however, they also said they had had higher expectations of this service because the staff at the pain centre was perceived to be experts in the field, and therefore they had hoped to receive treatment that would actually help them.

‘*I had expectations related to that they [the pain centre staff] had more experiences with pain and pain situations and that they therefore would be better to suggest different kinds of pain medication I could try out*’ (Male, 40- 44 years, attended treatment over time).

Other informants discussed more general expectations related to how to manage pain in everyday life, help to move forward and to be educated regarding what happens in their bodies when they experience pain. Some had expectations related to pharmacological treatments, including informants who hoped for a prescription for medical cannabis, or specific procedures such as nerve blocks, and others hoped to receive a diagnosis. For instance, one informant stated that she had frequently seen her GP over the last six years without receiving a diagnosis that could explain her pain.

‘*I have tried several services and medical tests. So, the pain centre became the last opportunity for both me and my GP*’ (Female, 25- 29 years, attended treatment over time).

### Experiences from attending the pain Centre

The pain centres’ multidisciplinary approach was new to most of the informants. Some said they found the approach useful as it led to all aspects of life with pain being considered. Several informants said they appreciated the focus on symptoms rather than on their diagnosis, which was different from other services they had received due to pain.

‘*On the whole, it was nice that at the pain centre they focussed on what actually was present. A kind of- this is the problem-, we do not care what causes it, we will just try to solve it the best way we can’* (Male, 40- 44 years, attended treatment over time).

While most informants appreciated the multidisciplinary approach, others found it unnecessary, as they did not recognise a need to be seen by all the attending professions. This could be because they simply wanted to receive a prescription, which required a consultation with a physician with a speciality in pain treatment. One informant stated that she had experienced the multidisciplinary session as a long interrogation in which the psychologist focused on previous life events related to her childhood and her broken marriage, even though her concerns were related to neuropathic pain.

‘*What I reacted to was that the physiotherapist said nothing, and the physician said next to nothing. What I felt they were fishing for were issues concerning my psyche. It was quite uncomfortable to put it mildly*’ (Female, 65- 69 years, attended one or two multidisciplinary sessions).

Several informants spontaneously discussed that at the pain centre, they had been warmly met and that this had left them with a feeling of being seen as a whole person. They said they had been listened to when presenting their struggles and questions, not needing to try so hard to explain themselves because it was obvious to everyone why they were there. The staff was perceived as calm, understanding and respectful which the informants said was reassuring. To some degree, this contrasted with other services they had attended in which some informants discussed being ‘looked down on’ when they sought help for their pain.

‘*There was nothing they [the pain centre staff] could do really because of my situation. But I experienced it [the treatment at the pain centre] as nice because they sincerely tried to suggest things I could try. I got to try out some medications as well. So, I think it was quite good’* (Female, 35- 39 years, attended one or two multidisciplinary sessions).

On the other hand, some informants had not experienced the meeting with the pain centre staff as positive. One informant said she perceived the staff to be discouraged with the work, and some were even rude and patronising. She said that the staff did not behave the way they should in meetings with a vulnerable group. For instance, when she had asked about the purpose of the multidisciplinary approach, she said she did not get any good answers, and consequently, she found the whole treatment approach unclear. Other informants also said it was unclear what the aim of the treatment was, which in turn made it difficult to determine when they had reached their treatment goals.

Among those offered only one or two multidisciplinary sessions, some said they found this to be a satisfying and adequate procedure, while others perceived it be too short and insufficient. One reason given was that they needed more time to get to know the staff to be comfortable with sharing their problems. They had wanted the treatment to proceed beyond the multidisciplinary sessions and said they found the procedure not to have room for adjustments to fulfil their specific needs for treatment. Instead, they were offered something they recognised as a standard procedure. One informant described the pain centre session as follows:

‘*They just ask about how you are doing, and I got the impression that they were checking your psyche. Whether your psyche was causing the pain and why you were there. The physiotherapist just measured things and the physician just asked questions*” (Female, 50- 54 years, attended one or two multidisciplinary sessions).

Other informants had received treatment over time. One example was having several sessions with the pain centre physician to try different pain medications to find the ones most helpful. For one informant, this gave confidence in that he had tried all available medicine. Another informant discussed follow-up between sessions with regular phone calls from the pain centre nurse. This gave him an opportunity to ask questions and to receive confirmation on whether he made the right choices for his treatment plan. He said this was reassuring and made him feel safe in his daily decision making. Another informant who had regular sessions over time with both the pain centre’s psychologist and the physiotherapist found them to fulfil each other as a team, although they provided separate sessions. She said the treatment at the pain centre was a lifebuoy in what for her was a demanding and exhausting life situation:

‘*It’s a really nice kind of lifebuoy coming here, when one has nothing else*’ (Female, 30- 34 years, attended treatment over time).

Most informants had attended a closing session during which a physician, a psychologist and a physiotherapist had been present. Some informants described the closing session as a session during which they were presented with a summary or a report the professionals had completed together beforehand. Although they were given the opportunity to provide input for the summary, it was said that the conclusions were perceived as already drawn. As such, some perceived the closing session as a briefing regarding what was decided by the staff rather than a session, they could actively participate in.

‘*They asked questions repeatedly, and I answered. Then, they had a conversation among themselves before I got back to them again. There was nothing else. I did not get any advice or something like that. It was a waste of time, really*’ (Female, 50- 54 years, attended one or two multidisciplinary sessions).

On the other hand, other informants said they found the closing session to be useful as it had presented advice on how to manage their pain even better. For some, this was a positive and even unexpected benefit, as they had not received this from any other service they had attended. For some informants, bringing their spouses to the closing session was suggested. Those who did said it was useful that the spouse received information about pain and pain management, which they expected to be a form of support later.

None of the informants had experienced that the questionnaire they had completed on beforehand had been part of their sessions. One of the informants said he found the questionnaire difficult to complete because his pain situation changed during the day, which made it difficult to provide a precise and correct score.

### Changes after attending the pain Centre

When asked about changes due to the pain centre treatment, some informants said they now perceived their days to be more stable with fewer pain fluctuations and that it was easier to work on accepting the pain after being told that their condition was not life threatening. One of the informants discussed coming to terms with his situation after being at the pain centre:

‘*In the end you just stop thinking that this day will be better than yesterday, but that is not a bad thing, really*’ (Male, 40- 44 years, attended treatment over time).

Some informants discussed positive changes related to the pain centre treatment, and one given reason was that it had served as a first step towards an improved everyday life. One informant said that if she had attended the pain centre earlier, she would have been disappointed because she then would have expected the treatment to cure her. Having experienced that previous treatment had not helped, made her appreciate the treatment helping her manage the pain better. Another informant said that at the pain centre he had received useful information regarding how both the body and the psyche affected his pain experience. He found this highly useful and had accordingly changed his work situation and included a focus on issues such as stress reduction, proper breathing and sleep into his everyday routine.

Some informants said that when reflecting on the pain centre, they believed they could have managed without it. Some said that attending the pain centre had been okay but also unnecessary because all they had been told was that they should just proceed with what they were already doing. One informant said the advice was frustrating because she had wanted to attend the pain centre because nothing she did, helped. Some informants said that because they had not received any information or advice that was useful, nothing had changed in their lives after the visit to the pain centre.

‘*I have been to my GP afterwards. We decided to just continue as before*’ (Female, 50- 54 years, attended one or two multidisciplinary sessions).

Several informants said they still searched for efficient pain treatment as their everyday life had not improved in any way; however, it was difficult to determine where to turn for reliable guidance and advice, and they said they would have appreciated it if the pain centre would have guided them in this process. Consequently, several informants said they lacked guidance regarding where to seek reliable and trustworthy information and advice regarding their situations:

‘*There is a lot of information out there. What is difficult is to know what to trust*’ (Male, 40- 44 years, attended treatment over time).

Some informants also said they would have appreciated being referred to a service that would build on what they had learned at the pain centre, such as in their municipalities. Some stated that it had been suggested to continue treatment, such as with a physiotherapist, but that it was sometimes difficult to find available and affordable treatment.

## Discussion

Experiences from attending a pain centre varied from the pain centre ‘being a lifebuoy’ by informants who had attended treatment over time, to being described as ‘a waste of time’ by others who only had attended one or two multidisciplinary sessions. As such, the experiences of attending a pain centre were more linked to the amount of treatment than to which of the pain centres they had attended. Overall, the majority of informants had a positive experience, such as meeting with professionals who understood and showed an interest in their situations. Few informants had expectations of the pain centre in general, and few expected treatment that would eliminate pain, partly due to little knowledge regarding what the treatment implied and partly due to negative experiences from previously attempted treatments.

### Varying experiences

The main finding was the large variation in the experiences of attending a pain centre. One variation was linked to the overall impression of attending the pain centre, ranging from perceiving it as ‘a lifebuoy’ to a ‘waste of time’. Positive experiences included being met with understanding and the perception of receiving the best possible treatment, but there were also negative experiences, including disappointment due to not being offered treatment exceeding the initial multidisciplinary assessment and a lack of benefits afterwards. Substantially different experiences from multidisciplinary pain treatments have also been found by others. One study showed that the overall impact of an interdisciplinary chronic pain intervention programme could be placed along a spectrum from whole life change to no change [22], whereas another study used the labels ‘overall life changes’ and ‘stagnation’ [27] to describe different experiences of a multimodal cognitive chronic pain treatment.

Another variation concerned the length, type and dosage or amount of treatment. Some found the amount of treatment to be just right, whereas others found it to be too short. Yet others discussed receiving treatment they had not perceived a need for, such as the presence of a psychologist at their sessions. Others have written about the difficulties of finding the optimum dosage of multidisciplinary pain rehabilitation programmes [28], concluding there is little evidence to support clinicians in decisions regarding the optimum dosage [28, 29]. Furthermore, different choices about what dose to provide have been found between different pain centres, implying that to a large extent, dosage is determined on a historical basis and according to available clinical expertise [29].

A third variation was related to the impact on the participants’ everyday life after having attended the pain centre. Some informants described important changes and improvements, while others said nothing had changed and that they simply had proceeded as before. This is in line with statements that only a 30% reduction in pain is achieved in about half of treated patients [10]. Single studies have reported improvement by embedding learned strategies and techniques [20] and increased self-understanding [30], but disappointment has also been reported due to the pain treatments’ focus on coping tools and relaxation methods [9].

### Meeting patients’ expectations

One explanation for the variations in experiences could be due to different expectations. A study from England showed that the pain centre staff often misjudged what was important to patients [31]. Thus, information related to actual possibilities and clarifying expectations prior to treatment seems important.

In the current study, informants stated that they completed a questionnaire before attending the pain centre but that their answers were not discussed during their sessions. This appears to be a missed opportunity to discuss patients’ expectations towards treatment. It might be beneficial to assess both pain and psychological distress prior to treatment and to align the treatment with the patients’ assessed needs accordingly [32]. This could ensure both a better match between common clinical pictures and the content of treatment to improve the results of multidisciplinary pain treatments and could ensure a more patient-centred care approach [33].

### Need for a different approach?

The variations presented can be ascribed to the complexity that characterises the situations of persons with chronic pain due to the impacts of physical, psychological and social factors [13]. Nevertheless, it is important to ask whether the variations in experiences are also related to how these persons’ needs are met at pain centres. Based on the informants’ responses, it seems that for many, the pain centre had provided what they considered a small amount of input for only a short period of time. Persons referred to pain centres have typically attempted several approaches that had not helped [9]. When not even a highly specialised tertiary service succeeds in meeting the needs of this varied group of persons in a meaningful way, a different approach could be warranted.

One of the most striking experiences described by the informants concerns that they feel left alone after discharge, and these experiences are in line with descriptions from studies on chronic pain treatment provided by other healthcare services [34]. With the chronicity as well as the fluctuating symptoms persons with chronic pain have [35], it is clear that they might need support at irregular intervals over a long period. The question arises regarding which role the pain centre should have in this support and whether they should be open to being contacted when the patients and their everyday providers feel they are experiencing the worst pain trajectory.

It could be argued that a highly specialised healthcare service such as a pain centre, should contribute to a patient’s pain treatment only for a short period, transferring the follow-up responsibility to primary care and a GP; however, dissatisfaction with the quality of pain treatment in primary care, including GPs, has been reported [36] along with challenges with continuity of care throughout the health care systems for this group of patients [37]. In addition, it has been stated that it is vital that primary care practitioners have access to timely and appropriate multidisciplinary resources to support the health and well-being of their patients [38]. Consequently, it could be argued that pain centres, with their multidisciplinary teams, should be a service available to patients and their day-to-day providers at irregular intervals over time to optimise the efforts they initiate.

### Strength and limitations

A strength of the study is the novelty in the exploration of experiences of persons attending outpatient individual multidisciplinary pain treatment that was not part of inpatient, group based or time-limited programmes, as in e.g., [22, 29, 30]; however, there are some noteworthy limitations. The aim was to obtain data that represented the variations in patients’ experiences in Norwegian pain centres in order to answer the research question. In line with Malterud, our aim was not to head for a complete descriptions of all aspects of patients experiences in the pain centres, but to offer new insights that can contribute substantially to or challenge current understandings [39]. The sampling strategy could have led to a biased sample as the informants were initially identified by the pain centre staff. Nevertheless, the sample showed variations as planned, including informants of both genders at different ages, who had received treatments at different lengths and with a diversity of experiences from the different pain centres. To minimize potential biases during the analysis, preliminary results were discussed with an extended research group to expose the data to different views and perspectives.

## Conclusions

For some, receiving a specialised multidisciplinary pain treatment represented a new approach that provides new insights into how to adjust to living with chronic pain: however, health professionals at the pain centres should recognise that some might experience the treatment procedure as insufficient and too short or even as overtreatment, and thus to not correspond with their specific and actual needs. Efforts should be devoted to ensuring that the pain centres’ multidisciplinary treatment approach is aligned with their patients’ actual needs.

## Supplementary information

**Additional file 1.** Interview guide.

## Data Availability

The raw data supporting the findings of the manuscript can be found at the Department of Public Health and Nursing, Trondheim, Norway. Due to regulations of the NSD, we must secure the anonymity of the informants. In the raw data, it is possible to identify the informants, and restrictions therefore apply to the availability of these data.

## Abbreviations

GP: General Practitioner
IASP: The International Association for the Study of Pain
