# Supplementary material for: ‘A lifebuoy’ and ‘a waste of time’: patients’ varying experiences of multidisciplinary pain centre treatment- a qualitative study
Source: BMC Health Serv Res. 2019 Dec 30;19:1015. doi: 10.1186/s12913-019-4876-5 (PMC6936064; doi:10.1186/s12913-019-4876-5)
Supplement: Supplementary file 1 — Additional file 1. Interview guide. [file 12913_2019_4876_MOESM1_ESM.docx]

# Interview guide

Age:
Education:
Profession:
Work situation:
Marital status:
Living situation:
Pain Duration:
Pain cause / type:
When finished treatment:

Main question:
- Can you talk about your experiences regarding the treatment you received at the pain centre?

Followed by questions on:

- What expectations did you have when the referral was sent to the pain centre?
- Briefly tell you about the treatment you received at the pain center.
- How do you feel now and what significance has the pain centre have in this?
- What are your thoughts about the future?
Keywords if needed: whether they found the pain centre different from other treatments they had attended, whether there were any changes in how they managed their pain and on their own participation and involvement in the treatment.
